# Noxious pressure stimulation demonstrates robust, reliable estimates of brain activity and self-reported pain

**DOI:** 10.1016/j.neuroimage.2020.117178

**Published:** 2020-11-01

**Authors:** Jade B. Jackson, Owen O'Daly, Elena Makovac, Sonia Medina, Alfonso de Lara Rubio, Stephen B. McMahon, Steve C.R. Williams, Matthew A. Howard

**Affiliations:** a Department of Neuroimaging, Institute of Psychiatry, Psychology & Neuroscience, King's College London, UK; b Wolfson Centre for Age-Related Diseases, Institute of Psychiatry, Psychology & Neuroscience, King's College London, UK; c MRC Cognition and Brain Sciences Unit, University of Cambridge, UK

**Keywords:** Noxious pressure, ICC, Test retest, Evoked-response fMRI, Pain, VAS

## Abstract

Functional neuroimaging techniques have provided great insight in the field of pain. Utilising these techniques, we have characterised pain-induced responses in the brain and improved our understanding of key pain-related phenomena. Despite the utility of these methods, there remains a need to assess the test retest reliability of pain modulated blood-oxygen-level-dependant (BOLD) MR signal across repeated sessions. This is especially the case for more novel yet increasingly implemented stimulation modalities, such as noxious pressure, and it is acutely important for multi-session studies considering treatment efficacy. In the present investigation, BOLD signal responses were estimated for noxious-pressure stimulation in a group of healthy participants, across two separate sessions. Test retest reliability of functional magnetic resonance imaging (fMRI) data and self-reported visual analogue scale measures were determined by the intra-class correlation coefficient. High levels of reliability were observed in several key brain regions known to underpin the pain experience, including in the thalamus, insula, somatosensory cortices, and inferior frontal regions, alongside “excellent” reliability of self-reported pain measures. These data demonstrate that BOLD-fMRI derived signals are a valuable tool for quantifying noxious responses pertaining to pressure stimulation. We further recommend the implementation of pressure as a stimulation modality in experimental applications.

## 1 Introduction

Since the advent of functional magnetic resonance imaging (fMRI), our understanding of the neural basis of nociceptive input has substantially increased (Apkarian et al., 2005; Tracey and Mantyh, 2007). Brain function has been investigated in states of both acute (e.g. Buffington et al., 2005; Vachon-Presseau et al., 2013) and chronic pain (e.g. Jensen et al., 2013; Lebel et al., 2008; López‐Solà et al., 2014), and has provided key insights into pain-related phenomena (e.g. Bogdanov et al., 2015; Sprenger et al., 2011), such as the placebo effect (Benedetti et al., 2005), offset analgesia (e.g. Derbyshire and Osborn, 2009), and conditioned pain modulation (Kennedy et al., 2016; Pud et al., 2009). The relationship between noxious input and pain perception has been extensively studied (e.g. Apkarian et al., 1999; Atlas et al., 2014; Bornhövd et al., 2002; Büchel et al., 2002). For example, signal changes in anterior cingulate, secondary somatosensory and insular cortices show a positive linear relationship to painful heat trials (Bornhövd et al., 2002; Büchel et al., 2002). More recently, a multi-level mediation approach similarly showed that these regions, amongst others such as inferior and superior parietal lobule, predicted variations in pain report (Atlas et al., 2014). Prominently, previous work has primarily utilised thermal stimuli to activate networks involved during nociceptive processing (e.g. Becerra et al., 2001; Becerra et al., 2004; Borsook et al., 2008; Brooks et al., 2005; Craig et al., 2000; Tracey et al., 2002). Other modalities, such as noxious pressure, have not been as extensively applied (Apkarian et al., 2005) but are becoming more frequent, particularly in clinical applications (e.g. den Boer et al., 2018; Ichesco et al., 2016). Notably, these aforementioned studies rely on the assumption that neural responses to pain are consistent over time, that is, that the recorded responses are reliable. However, pain is a multifaceted experience, resulting from an interplay of sensory, cognitive and affective aspects, and can therefore vary from one experience to the next. Furthermore, there is an overall low statistical power of fMRI studies across disciplines (Button et al., 2013). Thus, the robustness of the applied methodology, and its ability to effectively characterise pain responses in the human brain, requires further investigation (Borsook et al., 2011a; Borsook et al., 2011b; Mackey, 2013; Wartolowska, 2011).

Experience is not static over time, and pain intensity can fluctuate (Robinson et al., 2013). Despite variations over time, subjective measures can reliably capture the pain experience (e.g. Grafton et al., 2005; Hodkinson et al., 2013; Williams et al., 2000). For instance, a meta-analysis conducted on visual analogue scales (VAS), numerical ratings, and verbal rating scales showed high reliability for these measurements (Williamson and Hoggart, 2005). Comparatively, the robustness of noxious-induced brain activations is less clear (Bennett and Miller, 2010). A well-established measure of reliability, the intra-class correlation coefficient (ICC, 1979), has been the consistent method employed to quantify estimates of pain-induced blood-oxygen-level-dependant (BOLD) responses (Letzen et al., 2016; Letzen et al., 2014; Quiton et al., 2014; Upadhyay et al., 2015). ICC has been described in the context of consistency between ratings given by different judges; however, it is also used to assess the reliability of ratings across testing sessions and of imaging methods over time (Bennett and Miller, 2010; Caceres et al., 2009). ICCs for noxious thermal stimulation have been shown to range from “poor” to “excellent” (Fleiss et al., 2013) in pain-related regions (Letzen et al., 2014; Quiton et al., 2014; Upadhyay et al., 2015). Mechanical stimulation has also shown high repeatability in areas such as secondary somatosensory cortex, but lower and more variable repeatability in the primary somatosensory cortex and thalamus (Taylor and Davis, 2009). Altogether, more work is needed to determine the reliability of pain-induced imaging endpoints, and the robustness of noxious pressure has yet to be assessed, despite its increasing application in clinical paradigms.

In the present study, BOLD-fMRI was employed to examine pressure pain-induced brain responses, using an evoked-response paradigm. Test retest (ICC) of participants’ subjective pain ratings, and group-level, pain-induced BOLD signal responses were examined across two identical sessions. Next, to provide further information regarding pressure stimulation effects on the brain, a map was constructed to assess the effect size distribution across voxels for pain-induced responses. Finally, ICC analyses were implemented to determine the intra-subject inter-session reliability of the pain-induced neural endpoints.

## 2 Methods

### 2.1 Participants

Twenty-three healthy pain-free participants (nine females; mean age = 26 years, SD = 5.2) were recruited for the study. Two participants from the initial twenty-three were excluded from data analysis for not completing both sessions. All participants were right-handed [as assessed by the Edinburgh handedness inventory; (Oldfield, 1971)] with normal or corrected-to-normal vision, no history of neurological or psychiatric disorder or history of substance abuse and no MRI contradiction. Participants with a chronic pain condition, a history of hand/thumb trauma, or with a neurological condition affecting the hand were additionally excluded. Participants were asked in advance of the initial testing session whether they wore artificial fingernails and if they could be removed prior to taking part in the experiment. If removal was not possible, then these participants were excluded. Previous data has indicated that females exhibit variability in their pain responses due to the phase of the menstrual cycle (e.g. Iacovides et al., 2015; Martin, 2009; Teepker et al., 2010; Vincent and Tracey, 2010). Accordingly, female participants completed all three sessions of this study within the equivalent 10-day period of consecutive months (follicular phase; between day 1–10 of their menstrual cycle). Irregular menstrual cycles therefore constituted an exclusion. Further, to minimise the influence of diurnal variations on pain and BOLD signal (Hodkinson et al., 2014; Jiang et al., 2016), participants were always tested at the same time in the day. Moreover, participants were required to adhere to the following lifestyle guidelines; abstain from alcohol for 24 hrs and limit caffeine to a maximum of one caffeinated drink prior to each visit, abstain from nonsteroidal anti-inflammatory drugs or paracetamol for 12 hrs as well as the use of tobacco or nicotine containing products for four prior to each visit. Participants gave written informed consent and the experiment was approved by the Psychiatry, Nursing and Midwifery Research Ethics subcommittee at King's College London, UK (Ethics reference: RESCM-17/18–4769).

### 2.2 Procedure

Participants attended three separate sessions in total. The first session was a familiarisation and sensory thresholding session conducted in a mock scanner environment. The following two sessions were conducted in a MRI scanning unit and were identical for test retest purposes. The mean interval between each testing session was 10.8 days (SD 10.6).

#### 2.2.1 Session 1 (familiarisation and sensory thresholding)

At the commencement of the first session, participants underwent a Drugs of Abuse (DOA) test and breath alcohol test to assess for substance use and compliance with the study requirements. Furthermore, compliance with lifestyle guidelines was assessed. Next, participants underwent sensory thresholding for pressure stimulation. Each participant received one ascending series of pressure stimuli and one randomised series (for each hand separately). All stimulations were applied to the thumbnail using an automated, custom-made, pneumatic, computer-controlled stimulator with a plastic piston that applies pressure via a 1.13 cm^2^ hard rubber probe (Jensen et al., 2009; Jensen et al., 2010). The thumb was inserted into a cylindrical opening and positioned such that the probe applies pressure to the nail bed. The precision of pressure applied via the piston was calibrated over four repetitions to confirm reliability of the delivered force prior to commencing data collection for this study. This same pressure device was used for both the sensory thresholding session and for the evoked-pressure paradigm in the scanner for consistency.

In the first stage of the ascending series staircase, participants received stimulation at 55 kilopascals (kPA; 2 s duration) which increased incrementally in steps of 4 kPA (4 s intervals). Participants were required to inform the experimenter when they had reached their minimum pain threshold (first score > 0 on a pain scale made up of 100 elements, anchored with ‘no pain’ at one side (0) and ‘worst pain imaginable’ at the other side (100)). Participants were additionally asked to inform the experimenter when they reached their “high” pain threshold (first score = 70). These values were then used to compute the magnitude of five different pressure intensities within the range of each participant's minimum and high threshold, e.g. if the minimum pain threshold was represented by a pressure of 200 kPa in the ascending series and the high pain threshold = 70 was reached with a pressure of 600 kPa, the randomised series would consist of pressures of 200, 300, 400, 500 and 600 kPa. Each of these five stimulations were repeated three times, thus in total 15 stimuli of 2 s duration were delivered in a pseudo-randomised order at 24 s intervals. During the interval, participants were required to rate their level of pain using a button box in the contralateral hand on a computerised pain VAS (7 s duration total presentation). A first order polynomial function was used to determine each participant's representation of a score of 60, derived from the 15 ratings from the randomised series [for further details refer to Jensen et al., 2009]. This thresholding procedure was repeated for both the left and the right hand to account for differences in sensitivity between the left and right thumb.

#### 2.2.2 Session 2 and 3 (imaging acquisition)

**Fig. 1:**
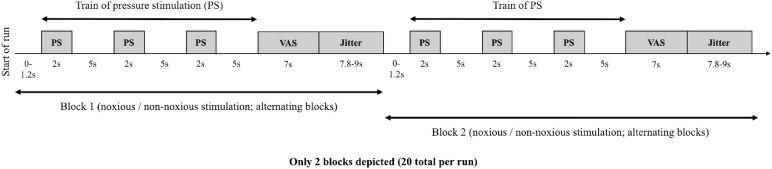
Evoked Pressure Paradigm Design. In each block (2 blocks depicted above) participants received a train of three pressure stimuli at either high (noxious; score of 60 determined by thresholding) or low (non-noxious; 55 kPA across all participants) intensity (alternating blocks). Within the train, each pressure stimulus had a total duration of 2 s with an interstimulus interval of 5 s. The first pressure stimulus occurred at a jittered interval after the start of the block (0–1.2 s). Following the train of pressure stimulation, participants were then presented with a computerised VAS of the pain scale. This scale was made up of 100 elements, anchored with 'no pain' at one side (0) and 'worst pain imaginable' (100). This scale was presented for 7 s. This was followed by a jittered interval prior to the start of the next block. There were 20 blocks of stimulation per run (10 noxious and 10 non-noxious blocks). Participants completed two runs in total (left- and right-hand stimulation). Each stimulus (within a train; noxious or non-noxious) contributed to the explanatory variable for either noxious or non-noxious stimulation (block dependant).

The procedure for the two imaging sessions was identical. Prior to entering the scanner participants underwent DOA and breath alcohol tests to assess for substance use and compliance with the study requirements. Furthermore, compliance with lifestyle guidelines was assessed. Next, participants underwent structural and localiser scans followed by the evoked-response pressure paradigm (Fig. 1), utilising the pressure probe previously described. The paradigm was a block design with alternating blocks of high (noxious: score of 60 determined on an individual basis by sensory thresholding in session 1) and low-pressure stimulation (non-noxious: average score of 8, 55 kPA, across all participants). Participants were informed that pressure stimulation would vary throughout the experiment; sometimes it would be higher and sometimes it would be lower. These instructions were non-specific so that participants were not aware of the thresholded values and further to reduce the likelihood of anchoring responses. The durations selected for event presentation were chosen for optimal design efficiency (Josephs and Henson, 1999). In each block (30.8 - 32 s duration) participants received a train of three pressure stimuli. Each pressure stimulus had a duration of 2 s. The first stimulus occurred at a jittered interval (0–1.2 s) after the start of the block enabling sampling of a different point in the participant's haemodynamic response when modelling the events. Each subsequent stimulus followed in intervals of 5 s. Succeeding each train of stimulation (three stimuli in total) and at the end of each block, participants were presented with a computerised VAS of the pain scale as in the thresholding procedure (7 s duration). This was followed by a blank black screen (jittered duration of 7.8–9 s) prior to the start of the next block. In entirety, there were 20 blocks of stimulation per run (10 noxious and 10 non-noxious blocks; total duration = 638 s). Participants completed two runs in total (one left- and one right-hand stimulation). Two separate runs were included in the experimental design (one per hand), and each hand was thresholded separately, to maintain a high total number of trials whilst minimising the effects of sensitisation.

### 2.3 Data acquisition

The data were collected using a 3T GE MR750 MRI scanner equipped with a 32-channel receive-only head coil (Nova Medical, USA) at the Centre for Neuroimaging Sciences, King's College London, UK. We used an echo planar imaging (EPI) acquisition sequence with the following parameters: repetition time (TR) 2000 ms; echo time 30; 48 slices with a thickness of 3 mm and a 0.3 mm inter-slice gap; matrix 64 × 64; field of view 211 mm^2^, flip angle 75°. Slices were acquired sequentially in descending order. High-resolution T1-weighted structural images were also acquired for all participants.

### 2.4 Preprocessing

MRI data were preprocessed using SPM 12 (Wellcome Department of Imaging Neuroscience, www.fil.ion.ucl.ac.uk/spm) in Matlab 2015b. Functional MRI data were converted from DICOM to NIFTI format, spatially realigned to the first functional scan (within session) and slice timing corrected. Translation and rotation parameters were determined to be in an acceptable range (< 2 mm and < 1.08° respectively for all participants). Structural scans were co-registered to the mean EPI and normalised, using the segment and normalise routine of SPM12, to derive the individual participant normalisation parameters. Normalised data were spatially smoothed (8 mm isotropic Gaussian kernel full-width at half maximum) to improve signal-to-noise ratio and were additionally high pass filtered (144 s).

### 2.5 Estimating bold signal responses to noxious stimulation

For the two primary conditions of interest (noxious and non-noxious stimulation; high and low pressure respectively) separate BOLD explanatory variables (EVs) were constructed. For both conditions (noxious and non-noxious), each of the trials began with a train of 3 stimulations, which were each modelled as having a duration of 2 s and an ISI of 5 s. Furthermore, for each condition, we constructed additional regressors encoding the period during which the VAS scores were collected. A final regressor was included for the blank black screen presented during rest, to minimise any superfluous noise in the model. All regressors were constructed separately for the two hands of stimulation. The resultant regressors were convolved with the canonical hemodynamic response function to produce ten BOLD EVs for modelling. Translation and rotation parameters (totalling 6 regressors), white matter and ventricular signal intensity were included in the model as covariates of no interest.

To establish the most robust effects of noxious pressure stimulation and incorporate all of our data, we first produced linear contrasts of parameter estimates (COPE) for each participant for the BOLD response to noxious stimulation compared to the implicit resting baseline (main effect of noxious stimulation; both hands of stimulation combined). We then generated two additional COPEs for each participant for each hand of stimulation. Next, COPEs were generated for each participant for the following comparisons: Noxious > non-noxious stimulation (i) of the left hand plus that associated with right hand stimulation, (ii) of the left hand, (iii) of the right hand. To test for group-related responses associated with each of the 1st level contrasts of interest, one sample t-tests were carried out. The statistical height threshold was set to *p* < 0.001, family-wise error (FWE), Gaussian Random Field (GRF) corrected at the cluster level (*p* < 0.05). To provide further information on the pain-induced BOLD responses that were subsequently submitted for test retest reliability, we calculated the size of the effect at each voxel. Effect size calculations (Cohen's d) were performed for the central contrast of interest (main effect of noxious stimulation). Following previous work (Geuter et al., 2018), the effect size was computed at each voxel (_v_) as the mean COPE divided by the standard deviation (across all subjects; *Ө_v_* = *μ_v_*/σ*_v_*). Groupings of effect size were based on guidelines from Cohen (Cohen and DuBois, 1999).

### 2.6 ICC reliability

#### 2.6.1 Behavioural measures

VAS scores were entered into a two-way ANOVA, with factors *Stimulation Type* (noxious, non-noxious) and *Session* (session 1, session 2). Next, coefficients of variation (CVs; SD/mean) were calculated separately for noxious and non-noxious stimulation, in each session, in each individual. As CVs were not normally distributed, we performed a log transformation (Log10) prior to entering the data into a two-way ANOVA with factors *Stimulation Type* (noxious, non-noxious) and *Session* (session 1, session 2).

Test retest reliability of VAS self-report pain scores were calculated between session 1 and session 2 (intra-subject, inter-session; collapsed across left- and right-hand). To assess reliability, the ΔVAS scores (noxious – non-noxious) and ICC (3,1) were computed using SPSS v19.0 (SPSS Inc., Chicago, IL, USA). Following previous recommendations (Fleiss et al., 2013), ICC values were categorised accordingly: < 0.4 as poor, 0.4–0.59 as fair, 0.60–0.74 as good, and > 0.75 as excellent. While a value of 1.0 indicates near-perfect agreement between the values of the test and retest sessions, a value of 0.0 would indicate that there was no agreement between the values of the test and retest sessions.

#### 2.6.2 Reliability of bold signal in response to noxious stimulation

To systematically evaluate the neural test retest performance, inter-session intra-subject reliability was estimated using the third ICC (Shrout and Fleiss, 1979) denoted by *ICC(3,1) = ((BMS-EMS)/(BMS+(k-1)EMS));* where BMS is the between-target mean squares, EMS is the error mean squares, and k is the number of repeated sessions*.* All ICC values were calculated in MATLAB 7.1 (The Mathworks Inc.) and the locally-developed ICC toolbox (Caceres et al., 2009). Intra-subject reliability was calculated at three levels: the whole brain, the complete activation network and the activated regions of interest (ROI), using a voxel-wise *t-statistic* threshold of 4.5 [following Caceres et al., 2009]. The activation network was obtained using a one sample *t*-test for the first session (for each contrast of interest separately). Functional ROIs were obtained in a second level analysis and were FWE, GRF corrected at the cluster level (*p* < 0.05), and obtained using an initial voxel-wise height threshold of *p* < 0.001. The ROI masks were extracted using the MarsBar toolbox (Brett et al., 2002). The medICC is reported, which is the reliability measure obtained from the median of the ICC distributions within regions (Caceres et al., 2009).

## 3 Results

### 3.1 Behavioural data

**Fig. 2:**
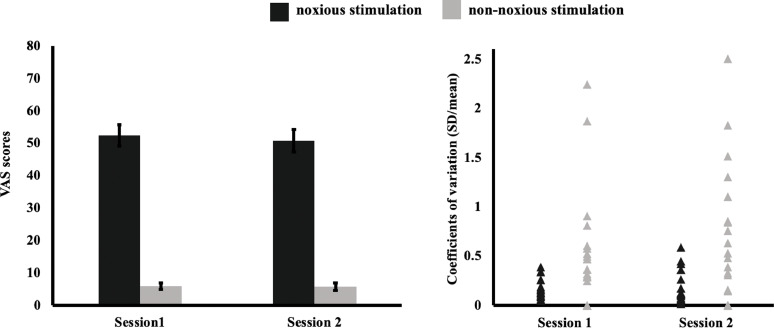
Behavioural Data. Left Panel: VAS scores (pain scale made up of 100 elements, anchored with 'no pain' at one side (0) and 'worst pain imaginable' (100)), were higher for noxious stimulation compared to non-noxious stimulation (ANOVA). No other main effects or interactions were significant. Error bars indicate standard error. Right Panel: Plot shows CVs by session and stimulation type (noxious, non-noxious). There was higher dispersion around the mean under non-noxious stimulation compared to noxious stimulation (main effect: ANOVA). No other main effects or interactions were significant.

VAS scores were entered into a two-way ANOVA with factors *Stimulation Type* (noxious, non-noxious) and *Session* (session 1, session 2). There was a main effect of *Stimulation Type* (F(1,20) = 271.5, *p* < 0.001) with higher VAS scores in the noxious (VAS; mean 51.1, SD = 14.7) compared to the non-noxious condition (VAS; mean 5.9, SD = 5.1). No other main effects or interactions were significant (all *p* values > 0.5). The VAS report is presented in Fig. 2 (left panel).

Next, we calculated CVs by stimulation type and session. A two-way ANOVA as above revealed a main effect of *Stimulation Type* (F(1,20) = 47.1, *p* < 0.001) with increased dispersion around the mean under non-noxious (CV; mean 0.57, SD = 0.61), compared to noxious stimulation (mean CV 0.17, SD = 0.13). No other main effects or interactions were significant (all *p* values > 0.4). Fig. 2, right panel, depicts CVs by *Stimulation Type* and *Session*.

Finally, the intra-subject inter-session ICC was obtained for our behavioural measures. An “excellent” degree of reliability was found for the ΔVAS (noxious – non-noxious) pressure scores between session 1 and session 2. The single measures ICC was 0.75 (95% CI [0.49, 0.89]).

### 3.2 Evoked responses to noxious pressure

The primary aim of the presented research was to calculate the reliability of BOLD signal responses pertaining to noxious pressure stimulation (incorporating the data from both left- and right-hand stimulation). We computed the main effect of noxious pressure (compared to an implicit resting baseline), to assess the ICCs of the pain-modulated signal over two identical sessions. For comparison, we additionally computed the reliability of noxious pressure against a baseline of non-noxious stimulation, as the baseline used for subtraction has previously been shown to modulate ICC estimates (Hodkinson et al., 2013). Therefore, in the following, we report data pertaining to both the main effect of, and the contrast of noxious stimulation.

**Fig. 3:**
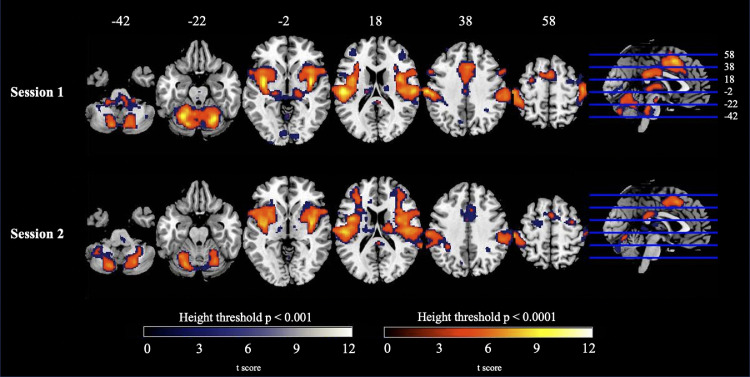
Evoked Activation for Main Effect of Noxious Pressure Stimulation (Data from Both Hands Incorporated) in Session 1 (Upper Panel) and Session 2 (Lower Panel). In session 1 there were clusters of activation in insula, thalamus and putamen extending into the postcentral gyrus. Additional regions included the cerebellum and primary somatosensory cortices extending into IFG. Peak activation in session 2 followed a similar pattern. Depicted here are two overlays at initial height thresholds *p <* 0.001 (blue to white), and *p* < 0.0001 (red to yellow).

Analysis of the main effect of noxious and non-noxious pressure stimulation (data from stimulation to each hand incorporated), at the recommended height threshold [*p* < 0.001; Eklund et al., 2016], revealed several large clusters reaching a size of 44,820 voxels. Accordingly, a more conservative height threshold (*p* < 0.0001) was adopted for these contrasts, in order to render these clusters interpretable (refer to Fig. 3 for a cluster extent comparison between the two height thresholds). At this more conservative threshold, a main effect of noxious stimulation (session 1 data) showed significant activity bilaterally across the insula, thalamus and putamen extending into the postcentral gyrus. Additional regions included the cerebellum and primary somatosensory extending into the inferior frontal gyrus (IFG), (refer to Table 1, upper panel, for full list of peak coordinates, and supplementary data for depiction of the size of the effect at each voxel for this contrast). In regard to the data from session 2 [main effect of noxious stimulation], there was a similar spread of activation across cortical and subcortical regions (refer to Table 1, lower panel, for full list of peak coordinates and Fig. 3; lower panel). The main effect of noxious pressure stimulation to each hand separately was additionally computed for comparison (refer to supplementary data). These data revealed a similar pattern to estimates of left- and right-hand stimulation combined, although, there was a strong degree of lateralisation with activation for right-hand stimulation dominating the left somatosensory cortices, and vice versa for left-hand stimulation.

**Table 1:** Peak Coordinates for Main Effect of Noxious Stimulation (Data from Both Hands Incorporated). Table lists peak coordinates for session 1 (upper panel) and session 2 (lower panel). The height threshold was set to *p* < 0.0001. Regions reported as bilateral (b), left hemisphere (l), or right hemipshere (r).

Main effect of noxious stimulation							
Session	Cluster	Peak coordinates			Cluster size	t	FWE (p)
		x	y	z			
1	thalamus/insula/putamen extending to postcentral gyrus (b)	−60	−20	46	8117	13.92	<0.0001
	cerebellum (b)	−26	−58	−26	6077	11.43	<0.0001
	postcentral/precentral gyrus extending into insula (r)	38	−4	−4	6761	11.02	<0.0001
	paracingulate gyrus (b)	−8	12	48	2009	9.27	<0.0001
	posterior cingulate cortex (b)	0	−30	26	689	7.28	<0.0001
	precentral gyrus (r)	34	−4	66	113	6.23	=0.011
	primary somatosensory extending into inferior frontal gyrus (l)	−58	12	34	119	5.89	=0.001
	intracalcarine cortex (r)	16	−72	8	78	5.33	=0.035
2	putamen/insula extending to postcentral gyrus (l)	−50	−20	22	6914	11.48	<0.0001
	putamen/insula extending to postcentral gyrus (r)	38	−6	−2	6407	9.34	<0.0001
	cerebellum (r)	24	−66	−48	751	9.33	<0.0001
	paracingulate gyrus (b)	4	16	50	1016	8.34	<0.0001
	cerebellum (l)	−32	−60	−48	1543	8.29	<0.0001
	cerebellum (r)	26	−66	−24	566	7.15	<0.0001
	thalamus (r)	16	−12	16	533	6.55	<0.0001
	posterior cingulate cortex (b)	10	−34	24	376	6.51	<0.0001
	thalamus (l)	−14	−22	10	241	6.50	=0.001

The main effect of non-noxious pressure (stimulation to both hands incorporated) demonstrated significant clusters of activation across several subcortical regions including the thalamus, insula and cerebellum (session 1). Additional regions included the postcentral gyrus, precuneus and visual cortical areas (lateral occipital complex and intracalcarine cortex), (refer to Table 2, upper panel, for full list of peak coordinates). For session 2, significant clusters included bilateral insula, postcentral gyrus, cerebellum, cingulate gyrus and IFG (Table 2, lower panel).

**Table 2:** Peak Coordinates for Main Effect of Non-noxious Stimulation (Data from Both Hands Incorporated). Table lists peak coordinates for session 1 (upper panel) and session 2 (lower panel). The height threshold was set to *p* < 0.0001. Regions reported as bilateral (b), left hemisphere (l), or right hemipshere (r).

Main effect of non-noxious stimulation							
Session	Cluster	Peak coordinates			Cluster size	t	FWE (p)
		x	y	z			
1	thalamus/insula extending to postcentral gyrus (l)	−52	−20	20	5887	15.71	<0.0001
	paracingulate gyrus (b)	4	10	48	1756	13.56	<0.0001
	insula extending into supramarginal gyrus (r)	60	−20	18	7346	11.03	<0.0001
	posterior cingulate cortex (b)	−4	−20	28	489	8.19	<0.0001
	thalamus (r)	10	−14	10	353	7.65	<0.0001
	cerebellum (l)	−12	−72	−42	116	7.50	=0.008
	precuneus (r)	4	−32	50	102	6.26	=0.013
	precuneus (b)	8	−68	40	326	6.23	<0.0001
	thalamus (l)	−12	−18	14	93	6.02	=0.018
	frontal pole (l)	−36	38	24	101	5.87	=0.014
	lateral occipital complex (l)	−56	−68	4	152	5.73	=0.003
	intracalcarine (r)	24	−50	−4	155	4.31	=0.003
	frontal pole (r)	42	38	10	152	4.29	=0.003
	intracalcarine (l)	−14	−66	4	140	5.51	=0.004
	insula (l)	−28	20	8	71	5.42	=0.039
2	insula extending to postcentral gyrus (r)	50	−16	24	3958	11.53	<0.0001
	insula extending to postcentral gyrus (l)	−52	−20	−22	4677	10.71	<0.0001
	cerebellum (l)	−16	−74	−42	175	8.63	=0.002
	paracingulate gyrus (b)	−6	12	48	586	7.30	<0.0001
	anterior cingulate gyrus (l)	−12	32	14	71	7.21	=0.046
	inferior frontal gyrus (l)	−32	−38	10	167	6.23	=0.003
	cerebellum (r)	24	−64	−48	130	5.59	=0.007

**Fig. 4:**
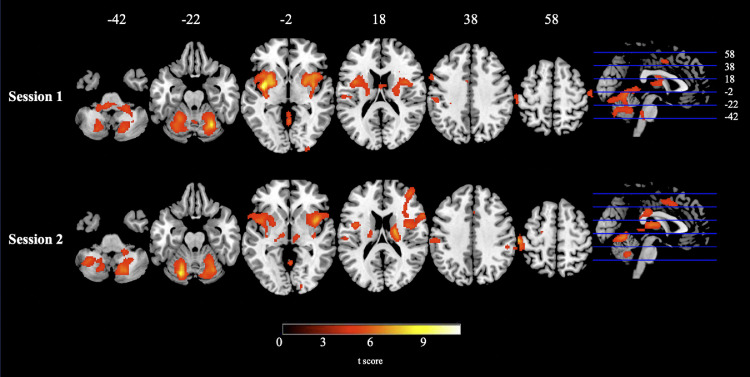
Evoked Activation for Noxious > Non-noxious Stimulation (Both Hands) for Session 1 (Upper Panel) and Session 2 (Lower Panel). In session 1 there were significant clusters of activation in regions including bilateral insula extending into the thalamus, putamen, and precentral gyrus. Additional regions included the cerebellum (bilateral) and primary somatosensory cortices. Peak activation in session 2 also centred in the thalamus, primary somatosensory cortices, cerebellar regions and precentral gyrus, and additionally in the IFG, cingulate and supramarginal gyrus. The height threshold was set to *p* < 0.001.

Paired *t*-test comparisons between noxious and non-noxious stimulation were additionally computed (data from both hands incorporated). For the contrast of noxious > non-noxious stimulation (session 1 data) there was significant activity in regions including the bilateral insula extending into the thalamus, putamen, and precentral gyrus. Further regions included the cerebellum (bilateral) and primary somatosensory cortices (refer to Table 3 for peak coordinates and Fig. 4; upper panel for session 1). Likewise, in session 2, peak activation for noxious > non-noxious stimulation was observed in the thalamus, primary somatosensory cortices, cerebellar regions and precentral gyrus, and additionally in the IFG, cingulate and supramarginal gyrus (Table 3 and Fig. 4; lower panel for session 2).

**Table 3:** Peak Coordinates for Noxious > Non-noxious Stimulation and Non-noxious > Noxious Stimulation (Data from Both Hands Incorporated). Upper region of table lists peak coordinates for session 1 and 2 [noxious > non-noxious]; lower region of table lists peak coordinates for session 1 and 2 [non-noxious > noxious]. The height threshold was set to *p* < 0.001. Regions reported as bilateral (b), left hemisphere (l), or right hemipshere (r).

Contrast	Session	Cluster	Peak coordinates			Cluster size	t	FWE (p)
			x	y	z			
N**oxious > non-noxious stimulation**	1	insula extending into the thalamus, putamen and precentral gyrus (b)	−38	−2	−4	5988	9.75	<0.0001
		cerebellum extending into the brain stem (b)	26	−62	−22	5633	7.94	<0.0001
		primary somatosensory cortex (l)	−50	−24	34	337	3.86	=0.021
	2	cerebellum (b)	−22	−72	−24	2199	8.15	<0.0001
		thalamus extending into right inferior frontal gyrus (b)	18	−8	16	4479	7.08	<0.0001
		primary somatosensory cortex (l)	−58	−24	58	897	6.82	<0.0001
		inferior frontal gyrus extending into thalamus (l)	−50	−2	8	1741	6.65	<0.0001
		cerebellum (r)	30	−54	−48	3108	6.54	<0.0001
		posterior cingulate gyrus (b)	8	−28	28	423	6.33	=0.011
		paracingulate gyrus (b)	12	14	48	291	4.01	=0.045
		precentral gyrus (l)	−32	−14	76	294	4.95	=0.043
		supramarginal gyrus (r)	50	−36	32	347	4.76	=0.024
N**on-noxious > noxious stimulation**	1	lateral occipital complex (r)	52	−54	14	5973	9.02	<0.0001
		precuneus (b)	−2	−38	68	2198	7.46	<0.0001
		postcentral/precentral gyrus (r)	36	−16	36	305	5.93	=0.03
		superior temporal gyrus (l)	−64	−8	−6	1265	5.77	<0.0001
		postcentral/precentral gyrus (l)	−36	−16	38	345	5.60	=0.019
	2	superior temporal gyrus (l)	−70	−22	−2	645	6.40	=0.001
		precentral/postcentral gyrus (b)	2	−36	70	930	5.91	<0.0001
		cuneus extending into lateral occipital complex (l)	−38	−80	32	777	5.55	<0.0001
		precentral/postcentral gyrus (r)	46	−8	32	321	5.38	=0.032
		precentral/postcentral gyrus (l)	−44	−10	32	296	4.78	=0.042

For the opposite contrast (non-noxious > noxious stimulation) there was significant activation in both sessions observed in occipital (e.g. lateral occipital complex) and temporal areas (e.g. superior gyrus) as well as in and around the postcentral/precentral gyrus (Table 3, lower panels for peak coordinates).

### 3.3 Test retest reliability of evoked noxious pressure

**Fig. 5:**
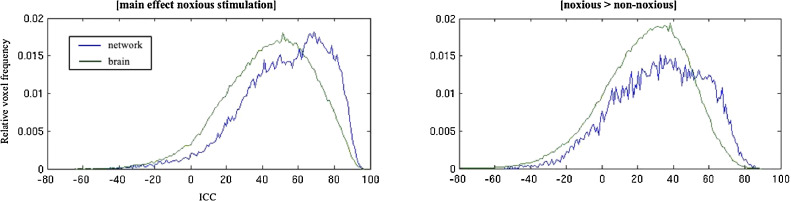
ICC Distributions. Plots show the relative number of activated voxels against ICC scores for the whole brain and activated network in response to evoked noxious pressure stimulation.

**Fig. 6:**
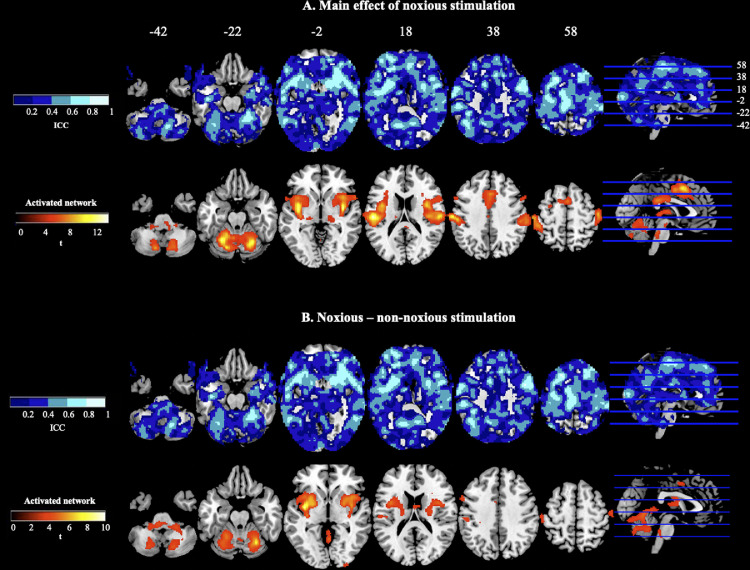
ICC Maps and Significant Clusters for Contrasts of Interest. 6A and 6B depict ICC values [dark blue; 0 to light blue; 1] on upper panels and significant clusters of activation [dark red to yellow, *t* scores] for contrast on lower panels. 6A. shows main effect of noxious stimulation and 6B. depicts the ICCs and contrast for noxious > non-noxious pressure stimulation.

ICC measures were implemented to examine test retest reliability of voxel-wise fMRI data. The results are presented in Table 4. For the main analysis of interest (main effect of noxious stimulation; stimulation to both hands incorporated), there was “fair” reliability in the brain (ICC: 0.46) and “good” reliability in the activated network (0.60). The relative number of voxels against ICC scores are plotted in Fig. 5 (left panel). Fig. 6A. (main effect of noxious pressure) illustrates ICC values across the brain in the upper panel, with significant clusters of activation from the pertinent second-level analysis in the lower panel. Significant clusters of activation from session 1 ranged between “poor” (lowest ICC = 0.33; intracalcarine cortex) to “good” reliability (highest ICC = 0.74; thalamus, insula, putamen, primary somatosensory cortices, IFG, and postcentral gyrus; refer to Table 4, upper panel). Reliability estimates were additionally computed for left- and right-hand stimulation separately for comparison to the composite of both (supplementary data). For the reliability of left-hand stimulation, significant clusters ranged from “poor” (lowest ICC = 0.04; thalamus) to “good” (highest ICC = 0.68; right primary somatosensory). For right-hand stimulation, clusters again ranged from “poor” (lowest ICC = 0.27; cerebellum) to “good” (highest ICC = 0.70; left supramarginal extending into postcentral gyrus). Moreover, reliability measures were computed for the main effect of non-noxious stimulation (Table 4, middle panel, for full list of ICCs). Comparative to reliability estimates for the main effect of noxious stimulation, ICCs were lower for both the brain (0.35) and activated network (0.52).

**Table 4:** ICC Coefficients. Upper panel pertains to the main effect of noxious stimulation, middle panel for the main effect of non-noxious stimulation, and lower panel pertains to the contrast of noxious pressure > non-noxious pressure (stimulation to both left- and right-hand incorporated). Regions reported as bilateral (b), left hemisphere (l), or right hemipshere (r).

Contrast	Region	ICC_m_	SE
M**ain effect of noxious stimulation**	brain	0.46	0.0007
	activated network	0.60	0.0018
	thalamus/insula/putamen extending to postcentral gyrus (b)	0.68	0.0022
	cerebellum (b)	0.34	0.0028
	postcentral/precentral gyrus extending into insula (r)	0.74	0.0019
	paracingulate gyrus (b)	0.56	0.0033
	posterior cingulate cortex (b)	0.43	0.0031
	precentral gyrus (r)	0.66	0.0117
	primary somatosensory extending into inferior frontal gyrus (l)	0.70	0.0065
	intracalcarine cortex (r)	0.33	0.0092
M**ain effect of non-noxious stimulation**	brain	0.35	0.0006
	activated network	0.52	0.0022
	thalamus/insula extending to postcentral gyrus (l)	0.61	0.0025
	paracingulate gyrus (b)	0.28	0.0058
	insula extending into supramarginal gyrus (r)	0.56	0.0027
	posterior cingulate cortex (b)	0.20	0.0008
	thalamus (r)	0.19	0.0123
	cerebellum (l)	0.34	0.0260
	precuneus (r)	0.36	0.0172
	precuneus (b)	0.04	0.0180
	thalamus (l)	0.23	0.0127
	frontal pole (l)	0.37	0.0123
	lateral occipital complex (l)	0.46	0.0063
	intracalcarine (r)	0.43	0.0140
	frontal pole (r)	0.47	0.0171
	intracalcarine (l)	0.33	0.0144
	insula (l)	0.50	0.0598
N**oxious > non-noxious stimulation**	brain	0.27	0.0006
	activated network	0.39	0.0032
	insula extending into the thalamus, putamen and precentral gyrus (b)	0.45	0.0047
	cerebellum extending into the brain stem (b)	0.25	0.0036
	primary somatosensory cortex (l)	0.45	0.0091

Reliability was also assessed for noxious pressure stimulation with a baseline measure (subtraction) of non-noxious stimulation for comparison. The relative number of voxels against ICC scores for the brain and network are plotted in Fig. 5 (right panel). For these data, there was poor reliability overall (brain; 0.27, activated network; 0.39) but “fair” reliability across a couple of the significantly activated clusters including a large cluster extending over the insula/thalamus/putamen and precentral gyrus (Table 4 and Fig. 6B).

## 4 Discussion

In the current study, we examined the reliability of acute noxious pressure, a now commonly implemented, but previously unassessed stimulation modality. Group-level analysis for noxious pressure, both the main effect of, and contrasted against non-noxious stimulation, revealed a number of regions of cortical and sub-cortical pain-related activation, in line with previous research (Apkarian et al., 2005). ICC calculations for the main effect of noxious pressure, which demonstrated large effect sizes, indicated good reliability across the activated network (0.60) as well as within significantly activated clusters (0.33–0.74). The reliability of the behavioural data was “excellent”, replicating previous findings of high reliability across behavioural measures (e.g. Bijur et al., 2001). These data inform our understanding on the nature of pain-induced BOLD signal establishing that pressure stimulation produces robust and reliable evoked-activation.

A substantial body of work has been conducted on the functional localisation of responses to noxious stimulation. For instance, a meta-analysis (Duerden and Albanese, 2013) of 140 neuroimaging paradigms revealed that whilst some activations are dependant on stimulus modality (e.g. heat vs. cold), the thalamus and insula are similarly activated regardless of the type of noxious stimulus, both of which activations were observed in the present report. In comparison, the number of reports that provide quantification regarding *test retest* ICCs of these pain-induced responses are sparse. Nonetheless, our findings echo previous investigations that have implemented ICC calculations of acute pain and demonstrated ranges of poor to excellent reliability (Letzen et al., 2016; Quiton et al., 2014; Upadhyay et al., 2015). The present study demonstrated that noxious pressure elicits high levels of reliability with “good” ICCs associated with regions commonly recruited during acute stimulation, including the insula, thalamus, putamen, IFG and somatosensory areas (e.g. Apkarian et al., 2005; Duerden and Albanese, 2013; Peyron et al., 2000). In these specific clusters, ICCs were observed in the range of 0.68 to 0.74, greater than the average report across disciplines (Bennett and Miller, 2010), and analogous to previous research that has examined the reliability of noxious heat. These prior studies reported coefficients within this range in the insula (Letzen et al., 2016; Quiton et al., 2014; Upadhyay et al., 2015), thalamus, inferior frontal regions, and somatosensory areas (Quiton et al., 2014; Upadhyay et al., 2015). This indicates that noxious pressure and heat have similar neural endpoints that are reliably activated over multiple sessions. However, as there is only limited data reporting ICCs of pain-induced responses, with some modalities yet to be assessed (e.g. noxious cold), future work is needed to determine the degree of stimulus-specific reliability.

In the present data we observed greater activation in the noxious compared to non-noxious condition in a wide range of regions including insula, thalamus, posterior cingulate and inferior frontal areas. However, high levels of reliability across the significantly activated clusters were only exhibited when scrutinising the main effect of noxious pressure. Comparatively, there was lower reliability across the activated network and significantly activated clusters when employing a baseline (subtraction) of non-noxious stimulation. These findings echo previous reports (Hodkinson et al., 2013) emphasising that the elected baseline plays an important role in measures of ICC. Here, a baseline of non-noxious stimulation does not provide a highly reliable endpoint, as stimuli are considered to be less salient and BOLD responses to non-noxious stimulation less stable across time. Note as well that within these reliability maps, as well as for those pertaining to the main effect of noxious stimulation, there were regions observed outside of the significantly activated clusters that displayed high levels of reliability. This has been previously demonstrated (Caceres et al., 2009), where highly activated regions have shown low reliability whilst some sub-threshold regions have displayed high reliability. It is not entirely unexpected that regions may convey a reliable BOLD signal without carrying significant information about the specified contrast. One reason for this being that fluctuations have been identified during both resting-state and active tasks that are believed to reflect long distance neural synchronisation (Buzsáki and Draguhn, 2004) and that are, in addition, reliable over time (Zuo et al., 2010).

The test retest characteristics of noxious BOLD-evoked responses are on a par with reliability reports from other sensory-motor, cognitive and affective domains. In a meta-analysis of fMRI test retest data (Bennett and Miller, 2010), reliability ranged from “fair” to “good” across all disciplines, with an average ICC report of 0.5. More recently a meta-analysis determined the average reported ICC at 0.4 (Elliott et al., 2019). In general, sensory and motor tasks tend to have high reliability. For example high ICCs are reported; 0.85 (Friedman et al., 2008), 0.76 (Kong et al., 2007) and 0.72 (Gountouna et al., 2010), for finger-tapping tasks. Comparatively, ICCs tend to be lower in the cognitive domain, such as in the case of reward-driven or n-back tasks [e.g. highest ICCs in ROIs 0.62, 0.57 respectively; (Plichta et al., 2012)]. These findings are broadly comparable to ICCs observed in this report for brain activity in response to noxious stimulation, and previous work in the field reporting ICCs > 0.7 (e.g. Letzen et al., 2014). However, comparing reliability data across investigations is not straightforward, not only in view of the relatively limited number of current ICC reports and modalities assessed, but also given methodological differences between studies in paradigm design, data acquisition and analytical approaches. For instance, Friedman and Glover (Friedman et al., 2008) showed that repeating the number of experimental runs between one and four in a sensory-motor task provided a positive linear increase in ICC, leading the authors to speculate that further repeats may continue to provide additional improvement. Other methodological factors including the test retest interval, sample size and design (e.g. blocked vs. event-related) will all play a role in reliability estimates.

It is important to consider the effects of inherent sources of noise in reliability estimates, such as variation due to motion, attention, and arousal (e.g. Cohen and DuBois, 1999; McGonigle et al., 2002). In this study translation and rotation parameters were assessed within-run and determined to be within an acceptable range. Scanning was also performed at the same time of day to minimise diurnal variation for each participant over repeated sessions (Jiang et al., 2016), and a constant level of arousal and attention was maintained by restricting caffeine consumption prior to scanning acquisition (Chen and Parrish, 2009; Liu et al., 2004); recommendations we would make for researchers considering similar studies. However, although participants received the same instructions in both sessions, it was not possible to fully control for expectancy and initial levels of saliency and anxiety, both of which have been shown to have a significant effect on an individual's level of pain perception (e.g. Baker and Kirsch, 1991; Brown et al., 2008; McGowan et al., 2009; Vase et al., 2005; Wager, 2005). Of note, however, is that participants’ first visits were conducted in a mock scanning environment to assist in minimising these effects. Nonetheless, it is a common experimental observation that anticipating, and being anxious about upcoming pain, can exacerbate the experience (Tracey and Mantyh, 2007). Therefore, one could speculate that a blocked design becomes predictable over two sessions (and thus initial anxiety, and saliency effects dissipate). In addition, as with repeated stimulation paradigms utilising visual stimuli (e.g. Parkes et al., 2004), noxious stimulation too decreases BOLD signal over repetitions (e.g. Bingel et al., 2007). However, whilst these aforementioned factors may have elicited variations over the two sessions, we utilised a blocked design as it provides maximal power, and significantly, despite the potential limitations of a blocked design, we observed high levels of reliability elicited by noxious pressure.

Intersession reliability may also vary based on the number of trials of painful stimuli delivered within-session. Here we employed a relatively large number of total trials, incorporating data from both left- and right-hand stimulation. When examining the estimates from each hand separately (effectively, utilising only half of the data), a similar spread of activation was observed, biased to the hemisphere contralateral to the stimulated side. Reliability estimates were only slightly lower for left- and right-hand stimulation considered separately. Whilst increasing the quantity of stimuli has the potential to increase power (Huettel and McCarthy, 2001) and intersession reliability, as observed here, it can additionally introduce fluctuations in BOLD response (Duann et al., 2002) that would add variability and decrease reliability as well as contribute to habituation or sensitisation.

The problem of response adaptation and habituation occurs in all sensory modalities (Thompson and Spencer, 1966), but is additionally difficult to avoid in experimental pain studies where participants are aware of the ethical responsibilities of the experimenter to ‘do no harm’ (https://www.iasp-pain.org/Education/Content.aspx?ItemNumber=1213). A core component of the pain response incorporates consideration of the potential threat to homoeostasis for the individual (Melzack, 2001; Moseley, 2003). Accordingly, only paradigms with moderate to severe evoked pain, as utilised here, are likely to persist over time and the robustness of observed brain responses to mildly or non-noxious stimulation may be reduced. That said, it remains an open theoretical question whether an appropriate isosalient non-noxious stimulus would demonstrate reliability characteristics more comparable with noxious stimulation. ‘Danger appraisal’ theories of pain (Moseley, 2003) suggest otherwise; pain motivates decision and action, with a greater priority to be processed compared to a highly-salient non-noxious stimulus (Wiech and Tracey, 2013), resulting in consistently strong responses over multiple sessions and higher reliability. However, we accept that the contrary viewpoint exists; as pain responses are dynamic and multifaceted, including varying levels of physiological arousal (Lee et al., 2020), variance estimates of responses to pain may be higher compared to a more uniform non-noxious stimulus, which may result in comparatively reduced reliability for noxious isosalient signals.

Although the reported data demonstrated high reliability for moderate-to-severe noxious pressure stimulation, it is a reasonable speculation that paradigms utilising the same stimulation modality with clinical populations may not elicit such highly reliable endpoints. In patients there may be further variations with regard to disease-specific pain and other related factors (e.g., fatigue, depression or frequency of medication), that can be present across time (Apkarian et al., 2011). To what extent these additional patient-specific fluctuations may impact on the reliability of the BOLD measures obtained is unknown. It is likely that background levels of spontaneous pain, a defining characteristic of chronic pain for many patients that waxes and wanes over time, introduces additional variability and thus lessens test–retest reliability of evoked pressure pain endpoints, compared to healthy controls. This is an important future consideration for experimental medicine research utilising pressure stimulation with an aim of developing brain-based biomarkers of acute and chronic pain states (Borsook et al., 2011a), and particularly in the case of ‘cross-over’ within-patient designs determining therapeutic responses (e.g. Svendsen et al., 2004).

In this work we assessed test retest reliability using a mass univariate framework, deriving ICC on a voxel by voxel basis. Voxelwise approaches have been extensively employed to derive mechanistic insights in how the brain responds to noxious stimulation (Brooks and Tracey, 2005; Neuroimaging, 2007). By contrast, multivariate ‘machine learning’ (ML) methodologies have been more recently employed that consider the contribution of all brain voxels in tandem. These approaches are appealing, as spatial correlations between activated voxels can be considered, providing potential improvements in sensitivity to detect experimental effects, for example, whether an individual is experiencing pain or whether a treatment may be effective. ML approaches also offer the desirable proposition to make predictions about new, previously unseen data, referred to as ‘generalisability’ (van der Miesen et al., 2019). They also offer great promise in the much-needed development of brain-based biomarkers for pain. To date, ML classification of experimentally induced pain in healthy volunteers have largely predominated, for example, in prediction of responses to thermal pain (Brown et al., 2011) as opposed to studies of real-world chronic pain states (van der Miesen et al., 2019). Further reports have demonstrated that classifiers could be specific to pain as opposed to other salient stimuli (Liang et al., 2013) and be able to detect modulation of pain response by analgesia (Wager et al., 2013)**.** Accurate generalisation performance of ML algorithms inherently requires a robust and unique ‘fingerprint’ of pain response that is detectable across individuals, both within and beyond the test sample under consideration. Ostensibly, these qualities bear similarity to assessment of test retest reliability; however, the reliability of ML pain classifiers, namely, the extent to which their predictions are consistent across time in each individual in the sample, remains to be put to proof. Given the potential for ML techniques to offer heightened sensitivity to detect pain, their reliability characteristics may accord with, or even exceed the current gold standard for investigating pain; participants’ own subjective reports. This is an important next step for ML technologies if they are to be exploited as diagnostic and prognostic markers for pain.

ICCs of self-reported pain indicated high reliability and were slightly higher than the most reliable fMRI brain responses. These findings accord with previous work stating high reliability for self-report of pain (e.g. Bijur et al., 2001; Gallagher et al., 2002; Hodkinson et al., 2013; Rosier et al., 2002; Williamson and Hoggart, 2005) also demonstrating self-report ICCs exceeding those associated with imaging endpoints (e.g. Letzen et al., 2014). In the present study, VAS inter-session ICC was 0.75, indicating excellent sensitivity to the changes in pain intensity within-subjects. If one were to view BOLD measures as a substitute for self-report, higher ICCs as compared to neuroimaging endpoints would be concerning. However, it is only through the use of a wide range of distinct methodologies that we will gain a greater understanding regarding behavioural and brain-based endpoints of pain. Self-report estimates such as VAS are one-dimensional and used in isolation do not adequately capture the multi-faceted experience of pain (Schiavenato and Craig, 2010; Williams et al., 2000). Further, ratings can be severely affected by cognitive factors, for example, social desirability bias (Van de Mortel, 2008). For example, it is possible that participants in this experiment actively attempted to rate consistently over the two sessions, potentially anchoring their responses to the two stimulation intensities. However note that to guard against anchoring behaviours, participants were purposely not informed that there would be only two stimulation types. Moreover, our observed CVs for VAS reports in noxious and non-noxious classes indicate moderate dispersion in ratings either side of mean VAS reports in each individual, suggesting against widespread anchoring behaviour.

Our VAS estimates were derived post-hoc and comprised a composite subjective report of three evoked stimuli. Whilst previous work has shown a close relationship in the mean and peak response of real-time pain intensity ratings to post-stimulus ratings (Koyama et al., 2004), post-hoc report is unlikely to fully capture the temporal dynamics of pain. However, this design choice was adopted with the intention of avoiding motoric and saccadic confounds on BOLD responses that would have been induced were participants to have rated continuously, which may have had an additional confounding effect on cross-session reliability estimates. Like many others, we suggest that neural and behavioural endpoints have different strengths and limitations but offer added value to one another when recorded in concert; the value in imaging pain is not to obviate self-report but to provide adjunct information.

Many factors can influence an individual's experience of acute pain over time. Here we have presented a test retest analysis of acute pressure stimulation across two fMRI sessions. ICC measures were implemented to quantify the reliability of both the brain and behavioural response to noxious pressure. The results indicate that noxious pressure elicits a reliable behavioural and pain-induced BOLD signal over two sessions. Moreover, stimulation by noxious pressure elicits activation across a vast range of regions previously shown to be fundamental to the perception of pain. These findings demonstrate that pressure stimulation is a viable method in the study of pain and are important for clinical research that is in the pursuit of developing biomarkers or that assumes reliability over repeated sessions, for example within-subject cross-over designs commonly adopted in the development of novel therapeutics.

## CRediT authorship contribution statement

**Jade B. Jackson:** Conceptualization, Formal analysis, Investigation, Writing - original draft, Visualization. **Owen O'Daly:** Formal analysis, Writing - review & editing. **Elena Makovac:** Conceptualization, Formal analysis, Writing - review & editing. **Sonia Medina:** Conceptualization, Investigation, Writing - review & editing. **Alfonso de Lara Rubio:** Software, Writing - review & editing. **Stephen B. McMahon:** Conceptualization, Writing - review & editing, Funding acquisition. **Steve C.R. Williams:** Conceptualization, Writing - review & editing, Funding acquisition. **Matthew A. Howard:** Conceptualization, Formal analysis, Writing - review & editing, Supervision, Funding acquisition.

## Appendix Supplementary materials

Image, application 1
