# Supplementary material for: Noxious pressure stimulation demonstrates robust, reliable estimates of brain activity and self-reported pain
Source: Neuroimage. 2020 Nov 1;221:117178. doi: 10.1016/j.neuroimage.2020.117178 (PMC7762811; doi:10.1016/j.neuroimage.2020.117178)
Supplement: Supplementary file 1 [file mmc1.docx]

**
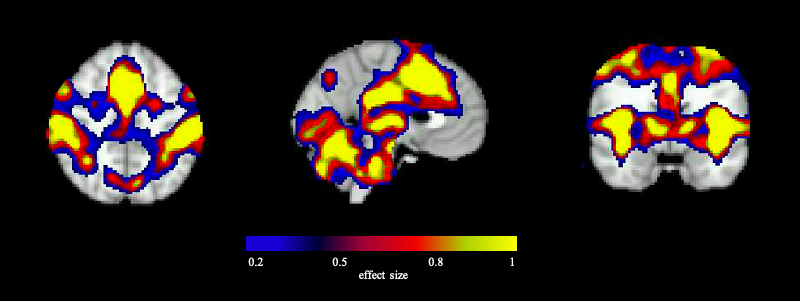
**

**Supplementary Figure 1: Effect Size Calculations for the Main Effect of Noxious Stimulation.** We implemented Cohen’s d computations at each voxel as the mean contrast of parameter estimate divided by the standard deviation (across all subjects). The figure depicts effect sizes ranging from small (blue; 0.2 – 0.5), medium (red; 0.5 - 0.8), to large (yellow; 0.8 - 1).


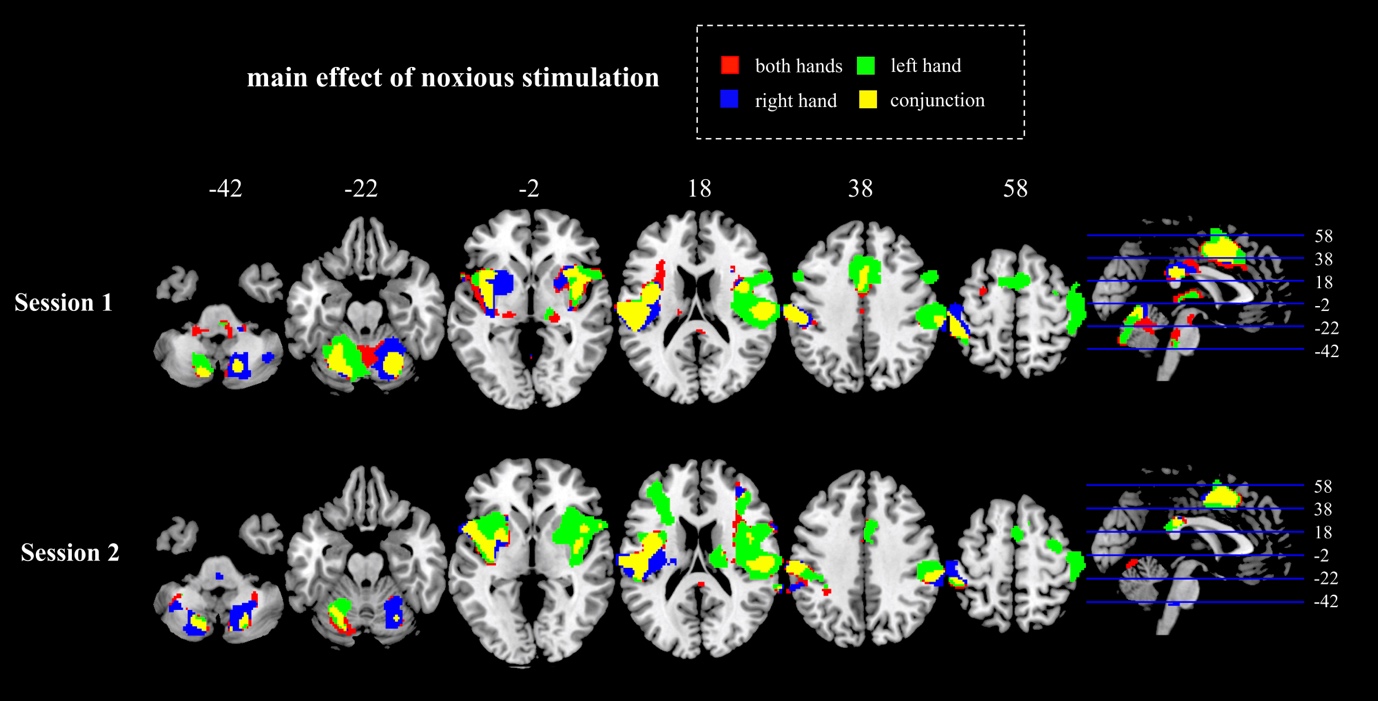


**Supplementary Figure 2: Evoked Activation for Noxious Pressure Stimulation for Session 1 (Upper Panel) and Session 2 (Lower Panel) for Stimulation Across Both Hands (Main Analysis)/Left-Hand Stimulation Only/Right-Hand Stimulation Only.** Yellow = conjunction; blue = right-hand stimulation only; green = left-hand stimulation only; red = stimulation to both hands incorporated (from main analysis). These data revealed a similar spread of activation for all main effects computed but emphasised lateralisation effects for stimulation to each hand when considered separately. The height threshold was set to *p* < 0.0001.

**
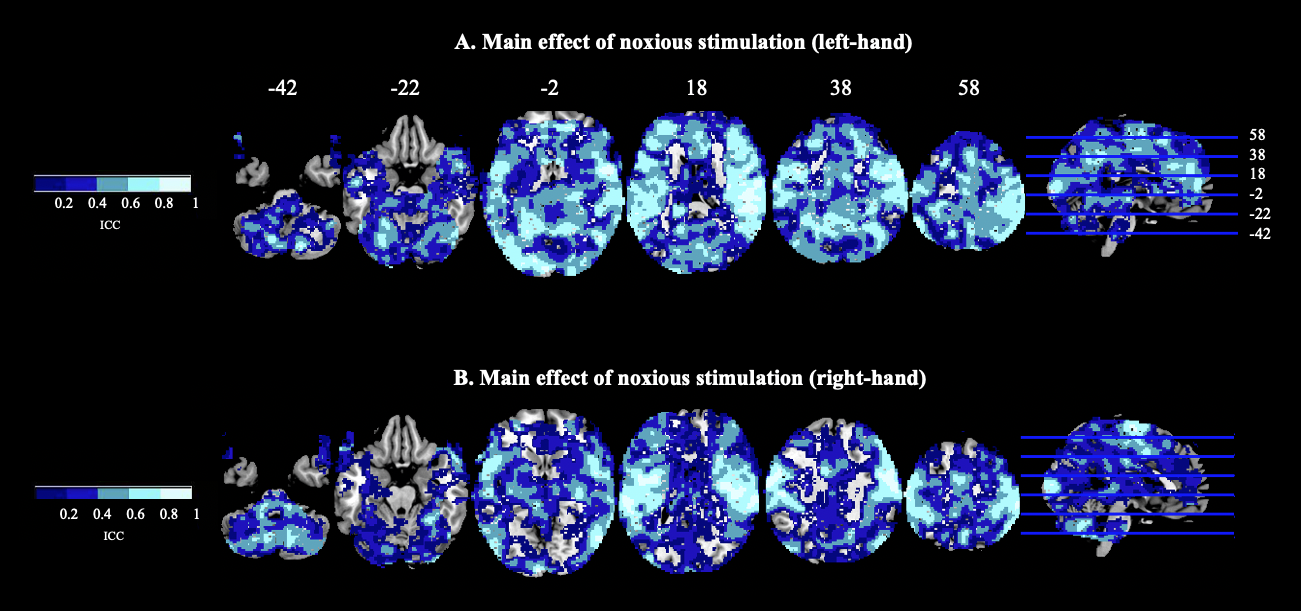
**

**Supplementary Figure 3: ICC Maps for Left- and Right-Hand Stimulation (Separately).** All figures depict ICC values [dark blue; 0 to light blue; 1]. Upper panel shows ICCs pertaining to the main effect of noxious stimulation to the left-hand only and the lower panel depicts ICCs pertaining to the main effect of noxious stimulation to the right-hand only. A similar spread is illustrated here as for the ICC data for stimulation to both hands incorporated together (Figure 6A, main manuscript).

| **Main effect of noxious stimulation (left hand only)** | | | | | | | |
| --- | --- | --- | --- | --- | --- | --- | --- |
| **Session** | **Cluster** | **Peak coordinates** | | | **Cluster size** | **t** | **FWE (p)** |
|  | | **x** | **y** | **z** |  | | |
| 1 | primary somatosensory (r) | 60 | -16 | 48 | 8198 | 11.43 | <0.0001 |
|  | postcentral gyrus (l) | -60 | 6 | 8 | 4602 | 11.02 | <0.0001 |
|  | cerebellum (b) | -28 | -58 | -26 | 3407 | 9.58 | <0.0001 |
|  | cingulate gyrus (b) | 0 | 18 | 44 | 2030 | 9.31 | <0.0001 |
|  | thalamus (b) | 14 | -16 | 10 | 846 | 7.75 | <0.0001 |
|  | inferior frontal gyrus (l) | -60 | 12 | 30 | 222 | 7.41 | =0.0001 |
|  | posterior cingulate gyrus (b) | -6 | -34 | 26 | 274 | 6.53 | <0.0001 |
|  | inferior frontal gyrus (r) | 40 | 40 | 8 | 113 | 6.25 | =0.010 |
|  | frontal pole (l) | -32 | 50 | 34 | 155 | 6.17 | =0.003 |
| 2 | primary somatosensory/insula(r) | 40 | -4 | 0 | 7433 | 10.66 | <0.0001 |
|  | postcentral gyrus/insula (l) | -52 | -20 | 22 | 5367 | 10.16 | <0.0001 |
|  | paracingulate gyrus (b) | 4 | 12 | 52 | 1226 | 8.28 | <0.0001 |
|  | cerebellum (l) | -32 | -52 | -50 | 1075 | 7.93 | <0.0001 |
|  | thalamus (r) | 18 | -18 | 18 | 341 | 7.39 | <0.0001 |
|  | posterior cingulate gyrus (b) | 8 | -38 | 24 | 288 | 7.30 | <0.0001 |
|  | cerebellum (r) | 24 | -64 | -48 | 212 | 6.73 | =0.001 |
|  | precentral gyrus (r) | 36 | -4 | 60 | 234 | 6.48 | <0.0001 |

**Supplementary Table 1: Peak Coordinates for Main Effect of Noxious Stimulation to Left-hand Only.** Table lists peak coordinates for session 1 (upper panel) and session 2 (lower panel). The height threshold was set to *p* < 0.0001.

| **Main effect of noxious stimulation (right hand only)** | | | | | | | |
| --- | --- | --- | --- | --- | --- | --- | --- |
| **Session** | **Cluster** | **Peak coordinates** | | | **Cluster size** | **t** | **FWE (p)** |
|  | | **x** | **y** | **z** |  | | |
| 1 | primary somatosensory/putamen/insula (l) | -56 | -22 | 48 | 6115 | 14.43 | <0.0001 |
|  | cerebellum (r) | 28 | -60 | -24 | 2350 | 11.66 | <0.0001 |
|  | cerebellum (l) | -24 | -62 | -22 | 678 | 9.60 | <0.0001 |
|  | supramarginal gyrus extending into postcentral gyrus (r) | 66 | -24 | 26 | 1068 | 9.54 | <0.0001 |
|  | putamen/insula (r) | 38 | 2 | -8 | 1391 | 9.25 | <0.0001 |
|  | paracingulate (b) | 0 | 0 | 44 | 716 | 7.36 | <0.0001 |
|  | anterior cingulate gyrus (b) | 2 | -28 | 28 | 336 | 4.68 | <0.0001 |
|  | thalamus (l) | -16 | -20 | 14 | 199 | 5.86 | =0.001 |
| 2 | primary somatosensory/putamen/insula (l) | -58 | -24 | 54 | 5005 | 11.05 | <0.0001 |
|  | cerebellum (l) | -24 | -76 | -40 | 918 | 8.99 | <0.0001 |
|  | cerebellum (r) | 20 | -62 | -46 | 786 | 8.24 | <0.0001 |
|  | cingulate gyrus (b) | 12 | 16 | 48 | 703 | 8.24 | <0.0001 |
|  | cerebellum (r) | 24 | -68 | -24 | 542 | 7.21 | <0.0001 |
|  | supramarginal gyrus extending into postcentral gyrus (r) | 64 | -20 | 24 | 1185 | 7.05 | <0.0001 |
|  | insula (r) | 40 | 4 | 12 | 377 | 6.44 | <0.0001 |
|  | frontal pole (r) | 34 | 42 | 14 | 110 | 6.08 | =0.015 |
|  | precentral gyrus (l) | -36 | -24 | 74 | 210 | 5.91 | =0.001 |
|  | primary somatosensory/putamen/insula (l) | 10 | -30 | 26 | 153 | 5.59 | =0.005 |
|  | cerebellum (l) | 56 | 8 | 12 | 261 | 5.55 | <0.0001 |
|  | thalamus (r) | 20 | -10 | 14 | 137 | 5.47 | =0.007 |

**Supplementary Table 2: Peak Coordinates for Main Effect of Noxious Stimulation to Right-hand Only.** Table lists peak coordinates for session 1 (upper panel) and session 2 (lower panel). The height threshold was set to *p* < 0.0001.

| **Contrast** | **Region** | **ICC_m_** | **SE** |
| --- | --- | --- | --- |
| **Main effect of noxious stimulation: (left hand)** | brain | 0.41 | 0.0007 |
|  | activated network | 0.55 | 0.0024 |
|  | primary somatosensory (r) | 0.68 | 0.0048 |
|  | postcentral gyrus (l) | 0.58 | 0.0028 |
|  | cerebellum (b) | 0.35 | 0.0045 |
|  | cingulate gyrus (b) | 0.43 | 0.0041 |
|  | thalamus (b) | 0.04 | 0.0057 |
|  | inferior frontal gyrus (l) | 0.57 | 0.0187 |
|  | posterior cingulate gyrus (b) | 0.36 | 0.0109 |
|  | inferior frontal gyrus (r) | 0.58 | 0.0141 |
|  | frontal pole (l) | 0.31 | 0.0169 |
| **Main effect of noxious stimulation: (right hand)** | brain | 0.26 | 0.0008 |
|  | activated network | 0.51 | 0.0032 |
|  | primary somatosensory/putamen/insula (l) | 0.63 | 0.0026 |
|  | cerebellum (r) | 0.29 | 0.0042 |
|  | cerebellum (l) | 0.27 | 0.0058 |
|  | supramarginal gyrus extending into postcentral gyrus (r) | 0.70 | 0.0050 |
|  | putamen/insula (r) | 0.52 | 0.0151 |
|  | paracingulate (b) | 0.45 | 0.0863 |
|  | anterior cingulate gyrus (b) | 0.31 | 0.0883 |
|  | thalamus (l) | 0.42 | 0.0892 |
| **Main effect of noxious stimulation: within-session across-hand** | brain | 0.34 | 0.001 |
|  | activated network | 0.35 | 0.002 |
|  | thalamus/insula/putamen extending to postcentral gyrus (b) | 0.43 | 0.004 |
|  | cerebellum (b) | 0.29 | 0.005 |
|  | postcentral/precentral gyrus extending into insula (r) | 0.35 | 0.004 |
|  | paracingulate gyrus (b) | 0.33 | 0.004 |
|  | posterior cingulate cortex (b) | 0.29 | 0.008 |
|  | precentral gyrus (r) | 0.11 | 0.016 |
|  | primary somatosensory extending into inferior frontal gyrus (l) | 0.44 | 0.012 |
|  | intracalcarine cortex (r) | 0.08 | 0.016 |

**Supplementary Table 3: ICCs for Left-, Right- and Within Session Between-hand Estimates.** Upper panel pertains to the main effect of noxious stimulation (left-hand only), middle panel for right-hand only, and lower panel to within-session between-hand reliability (run 1; left-hand to run 2; right-hand stimulation within session 1). The ICCs for left- and right- hand stimulation considered separately are within the range of ICCs for stimulation to both hands incorporated together, although slightly lower due to a decreased number of trials in the model. Within-session between-hand reliability shows qualitatively lower ICCs than other reliability estimates.
